# Ultra-Short-Term Corneal Changes to Nd:YAG Laser Capsulotomy: Energy-Dependent Changes Assessed by Specular Microscopy and Topographic Analysis

**DOI:** 10.3390/diagnostics15172280

**Published:** 2025-09-08

**Authors:** Çağrı Mutaf, Ali Hakim Reyhan, Mübeccel Bulut, Funda Yüksekyayla

**Affiliations:** 1 Department of Ophthalmology, Faculty of Medicine, Harran University, 63100 Şanlıurfa, Türkiye; alihakimreyhan@gmail.com (A.H.R.); f.dilmen@hotmail.com (F.Y.); 2 Department of Ophthalmology, Necip Fazil City Hospital, 63300 Kahramanmaras, Türkiye; mubeccelbagdas@gmail.com

**Keywords:** Nd:YAG laser capsulotomy, posterior capsular opacification, corneal endothelial cell density, anterior chamber parameters, energy-dependent changes

## Abstract

**Background:** This prospective observational study was conducted to systematically assess immediate changes occurring (within one hour) in corneal endothelial cell morphology and anterior segment parameters following Nd:YAG laser posterior capsulotomy in pseudophakic patients and to analyze the correlation between these changes and laser energy parameters. **Methods:** A single-arm, within-subject pre–post design was employed to evaluate corneal endothelial morphology (cell density, count, area, coefficient of variation and hexagonal percentage) and anterior chamber parameters (depth, angle, volume) before and one hour after the procedure using specular microscopy and Pentacam analysis. Patient demographics (age), clinical parameters (best corrected visual acuity and intraocular pressure), postoperative-YAG laser interval, and laser energy parameters (energy per shot, pulse count, and total applied energy) were also documented. **Results:** Thirty-two pseudophakic patients (mean age 56.3 ± 19.2 years) underwent Nd:YAG laser posterior capsulotomy with mean energy per shot of 3.15 ± 1.07 mJ and pulse count of 34.3 ± 20.4. Specular microscopy revealed significant post-procedural decreases in endothelial cell density (2184.05 to 2057.2 cells/mm^2^; *p* = 0.006) and increases in average cell area (529.25 ± 242.72 to 587.75 ± 281.09 µm^2^; *p* = 0.004) and minimum cell area (199.3 ± 170.62 to 248.35 ± 202.7 µm^2^; *p* = 0.035). Corneal topography also decreased significantly in the anterior chamber angle (40.07 ± 10.34 to 35.42 ± 6.78 degrees; *p* = 0.048), with positive correlations between energy per shot and endothelial cell density (r = 0.557; *p* = 0.011) and average cell area (r = 0.544; *p* = 0.013). **Conclusions:** This study demonstrates that Nd:YAG laser capsulotomy causes immediate, energy-dependent alterations in corneal endothelial density and anterior chamber parameters within one hour post-procedurally. The identification of energy per shot as a key determinant represents a preliminary observation for optimizing laser parameters and reducing potential complications in pseudophakic patients.

## 1. Introduction

Cataract surgery represents one of the most frequently performed surgical procedures worldwide, with millions of operations conducted annually to restore vision in patients with lens opacification [1]. The evolution of surgical techniques, particularly the development of phacoemulsification and advanced intraocular lens (IOL) technology, has significantly improved surgical outcomes and patient satisfaction. However, despite these advances, posterior capsular opacification (PCO) remains an inevitable long-term complication that affects a substantial proportion of patients following cataract extraction [2].

Commonly referred to as “secondary cataract,” PCO occurs when residual lens epithelial cells proliferate and migrate across the posterior capsule surface, leading to capsular fibrosis and visual impairment [3]. This biological process typically manifests months to years after the initial cataract surgery, with reported incidence rates varying from 20% to 50%, depending on patient demographics, IOL design, and surgical techniques employed [2,4,5,6]. The development of PCO significantly compromises visual quality, causing symptoms such as decreased visual acuity, glare sensitivity, and reduced contrast sensitivity, ultimately necessitating secondary intervention to restore optimal visual function [6,7,8]. Accordingly, to reduce the likelihood of PCO, intraoperative strategies emphasize meticulous cortical cleanup, a 360-degree capsulorhexis–optic overlap, secure in-the-bag IOL positioning, and the use of sharp-edged hydrophobic acrylic lenses, while in pediatric or other high-risk eyes, primary posterior capsulotomy and/or optic capture may be considered [9].

The Nd:YAG laser is widely employed for PCO management in ophthalmology. This laser system uses photodisruption principles, employing ultra-short pulses of infrared light at a 1064-nanometer wavelength to target the opacified posterior capsule with reduced thermal effects on surrounding tissues. Nd:YAG laser posterior capsulotomy represents the most effective treatment method for PCO, and is distinguished by its simplicity, efficiency, and minimally invasive nature [10]. The procedure is performed on an outpatient basis, involving a precise 3–5 mm central opening in the opacified posterior capsule that permits immediate visual rehabilitation and substantial improvement in quality of vision. However, photodisruption during Nd:YAG laser capsulotomy generates acoustic shock waves, cavitation bubbles, and debris particles throughout the anterior chamber.

Despite the efficacy of Nd:YAG posterior capsulotomy, early complications such as intraocular pressure (IOP) elevation, anterior segment inflammation, cystoid macular edema (CME), and IOL pitting have been reported. A recent prospective series involving 220 eyes reported an overall complication rate of 13.76%, with IOP rises being transient and manageable [11]. These physical phenomena, together with inflammatory mediator release and transient intraocular pressure elevation, create conditions that may adversely affect anterior segment structures and ocular homeostasis.

The corneal endothelium, a single-cell layer, represents a critical determinant of corneal transparency by regulating stromal hydration via barrier and Na+/K+-ATPase–mediated pump functions. Endothelial cell density (ECD) in adults is approximately 2500–3000 cells/mm^2^, declining by approximately 0.6% per year, accompanied by increased polymegathism (a higher coefficient of variation, CV) and reduced hexagonality (pleomorphism). Alterations in ECD and morphology quantified by specular microscopy are associated with stromal fluid accumulation, increased central corneal thickness, and reduced visual acuity. Due to their post-mitotic nature, these cells are susceptible to mechanical and hydrodynamic stress [12,13,14]. During Nd:YAG capsulotomy, acoustic shock waves and cavitation bubbles may propagate within the anterior chamber and can transiently disrupt endothelial homeostasis, potentially manifesting as changes in CV and hexagonality and, to a lesser extent, small short-term declines in ECD. It may therefore be prudent to adopt a lowest-reasonable-energy approach and early post-procedure specular monitoring, particularly in eyes with endothelial risk factors such as Fuchs endothelial corneal dystrophy or diabetes.

Specular microscopy serves as the gold standard for non-invasive endothelial assessment, providing detailed cell visualization and precise quantification of density, morphology, and functional parameters through high-resolution automated imaging. Corneal topography complements this evaluation as an essential diagnostic modality, using sophisticated imaging to create detailed maps of corneal curvature, thickness, and elevation, thus enabling comprehensive anterior segment assessment and detection of subtle structural changes. The integration of specular microscopy and corneal topography provides enhanced sensitivity for comprehensive assessment of laser-induced anterior segment changes.

Assessing the immediate corneal endothelial and anterior segment effects of Nd:YAG laser posterior capsulotomy is essential for patient safety and procedural optimization. While generally safe, energy-dependent endothelial damage and anterior chamber alterations may occur, particularly in compromised patients, thus necessitating systematic evaluation to prevent complications. This prospective observational study aimed to investigate the correlation between laser energy parameters and early morphological changes in corneal endothelial cells and anterior segment parameters following Nd:YAG laser posterior capsulotomy. Comprehensive endothelial morphology and anterior chamber measurements were evaluated at one hour post-procedurally using standardized specular microscopy and Pentacam HR corneal topographic analysis in pseudophakic patients.

## 2. Materials and Methods

This prospective, observational study was conducted at the Harran University Department of Ophthalmology, Türkiye, between April and July 2024. The study protocol was approved by the Harran University Clinical Research Ethics Committee (Decision No. HRÜ 25 June 2025, dated 24 March 2025) and complied with the tenets of the Declaration of Helsinki. Informed consent was obtained from all subjects involved in the study. This research employed a single-arm, within-subject pre–post design in which only one eye per patient underwent Nd:YAG laser capsulotomy.

The study population consisted of pseudophakic patients who had previously undergone uncomplicated phacoemulsification surgery with in-the-bag monofocal IOL implantation using Sensar hydrophobic acrylic single-piece lenses (Johnson & Johnson Vision, Irvine, CA, USA) and who subsequently developed clinically significant PCO. Nd:YAG laser capsulotomy was performed exclusively on eyes presenting with visually significant PCO requiring therapeutic intervention. Selection for treatment was based on clinical necessity rather than randomization.

Inclusion criteria were best corrected visual acuity (BCVA) ≤ 0.5 (Snellen equivalent), clear cornea with no pre-existing pathology, and ability to provide informed consent. Informed consent was obtained from all subjects involved in the study. Exclusion criteria encompassed previous ocular surgery other than uncomplicated cataract extraction, corneal pathology or dystrophy, glaucoma or ocular hypertension, diabetic retinopathy or other retinal pathology, uveitis or a history of intraocular inflammation, and inability to cooperate with examinations. Patients with a baseline endothelial cell density below 1500 cells/mm^2^ according to specular microscopy measurements were also excluded from the study.

In order to ensure reproducibility and minimize inter-operator variability, all Nd:YAG laser capsulotomies were performed by the same experienced operator (AHR), and all measurements were conducted by the same technician under standardized dim lighting conditions. All measurements were performed in triplicate and averaged to enhance accuracy and reliability.

### 2.1. YAG Laser System

The Quantel Medical Optimis II Nd:YAG laser system (Quantel Medical, Cournon d’Auvergne, France) operates at a wavelength of 1064 nm, with an energy delivery range of 0.5 mJ to 10 mJ per pulse, and a pulse duration of approximately 4 nanoseconds. The system features a spot size of 10 μm (with 86.4% energy in diameter) and a divergence angle of 8 μm (full width at half maximum). The repetition rate can be adjusted up to 15 Hz, with an offset capability of ±30 μm, ±100 μm, and ±200 μm, and pulse modes include single, double, and triple pulses per shot. The aiming beam is a red laser at 650 nm with adjustable intensity, and the slit lamp type employs a convergent optical path optimized for anterior and posterior segment viewing. The optical specifications maintain a spot size of 10 μm with 86.4% energy concentration within the diameter, while the laser’s cooling system maintains operation at room temperature. The laser is classified as Class 4 according to laser safety standards, with specific warnings for laser radiation exposure. Overall, the Optimis II system integrates advanced optical and electronic components to deliver precise, high-performance photodisruption for ophthalmic procedures.

### 2.2. Specular Microscope

Corneal endothelial cell morphological analysis was performed using a non-contact specular microscope (NCSM) (Nidek Co. Ltd., Gamagori, Aichi, Japan), with data collection focused on the central corneal region overlying the apex of the cone. Cell analysis was conducted by means of the automated analysis function integrated within the microscope system. Patients were positioned using the headrest and chin support, with forward gaze maintained on the fixation target. Images were captured after aligning the instrument with the corneal Purkinje reflex. The system photographed a 0.1 mm^2^ endothelial area, generating 16 images per session. The three best-quality images were selected for automated analysis. Specular microscopy provided the following morphometric parameters: cell count (NUM), endothelial cell density, coefficient of variation (CV), minimum cell area (MIN), maximum cell area (MAX), standard deviation (SD), hexagonal cell percentage (HEX), and average cell area (AVG).

### 2.3. Corneal Topography

Corneal topographic evaluation was performed using the Pentacam HR rotating Scheimpflug camera system (Oculus Optikgeräte GmbH, Wetzlar, Germany), a non-contact imaging device that provides comprehensive anterior segment analysis through 360° rotational scanning. The following parameters were measured and analyzed: anterior chamber depth (ACD), representing the distance from the corneal endothelium to the anterior lens surface; anterior chamber angle (ACA), measuring peripheral angles between the iris and corneal periphery; anterior chamber volume (ACV), quantifying the total volume of the aqueous humor space; and central corneal thickness (CT) at the corneal apex (apex CCT), measuring CT at the thinnest point. All patients were positioned with their chins on the chin rest and foreheads against the headband. They were then instructed to fixate on the central target light while the device completed automatic scanning, with multiple scans being obtained for each eye. Only examinations with acceptable quality scores were included in the analysis.

Pre-procedural pupillary dilation was performed using two applications of 1% cyclopentolate drops with a 15-min interval between them. A standardized waiting period of one hour following the initial cyclopentolate instillation was applied before commencing the Nd:YAG laser capsulotomy in order to ensure adequate mydriasis and cycloplegia. Comprehensive baseline measurements were obtained immediately before the laser procedure, including best corrected visual acuity, intraocular pressure measurement using an iCare tonometer (Revenio Group, Helsinki, Finland), autorefractometry with a Tonoref 3 autorefractometer (Nidek Co., Ltd., Gamagori, Japan), corneal topography using Pentacam HR, and specular microscopy assessment for both eyes. All baseline measurements were performed in triplicate under standardized conditions to ensure accuracy and reproducibility. The identical measurement protocol was repeated at one hour post-procedure to assess immediate corneal changes following Nd:YAG laser capsulotomy.

YAG laser posterior capsulotomy was performed using the circular technique exclusively in all cases. The initial energy was set at approximately 1.6–5 mJ and was increased in 0.2–0.5 mJ steps until capsule disruption was achieved with minimal fragmentation and bubble formation. Once this threshold was reached, the same energy level was maintained for the remainder of the procedure. The procedure involved systematic placement of laser shots in a 360° circular pattern with consistent per-case energy settings following titration, targeting a capsulotomy diameter of approximately 5 mm based on clinical assessment of posterior capsule opacity. This technique achieved a regular, round capsular opening with smooth edges. The laser operation was conducted in Q-switched mode, using a beam configuration with a cone angle of 30°. The minimum energy level sufficient to create a capsular opening for each patient was determined through initial test shots, and was then held constant throughout the entire capsulotomy procedure. Total laser energy (mJ), number of laser shots, pulse energy per shot, capsulotomy diameter, and any intraoperative complications were meticulously documented for each case. The total cumulative laser energy delivered to each patient was calculated using the formula E_total = E_shot × N_shots, where E_total represents the total energy delivered (mJ), E_shot denotes the energy per individual shot (mJ), and N_shots indicates the total number of shots administered during the procedure. The laser parameters remained constant throughout each individual procedure to ensure consistency and reproducibility of the treatment protocol.

Post-procedural anti-inflammatory management consisted of dexamethasone 0.1% eye drops (Maxidex, Alcon) administered four times daily for five days to the treated eye only, with patient monitoring for potential complications. A one-month follow-up protocol was implemented to monitor all patients for post-procedural complications, with systematic evaluation of potential adverse events related to the Nd:YAG laser capsulotomy procedure. Only patients with no procedure-related complications were included in the final analysis.

The primary outcome measure was the correlation between total laser energy and early corneal changes at one hour post-procedure, specifically changes in endothelial cell density and central corneal thickness (CCT) from baseline measurements. Secondary outcomes included pre- to post-procedural changes in corneal topographic parameters, changes in specular microscopy parameters, and correlations between number of laser shots and magnitude of corneal changes.

### 2.4. Statistical Analysis

Statistical Package for Social Sciences for Windows version 27.0 software was used for statistical analyses. Descriptive statistics for continuous variables were expressed as mean and standard deviation, and descriptive statistics for categorical data as frequency and percentage values. Pre–post continuous variables were compared using the Wilcoxon signed-rank test, and effect sizes were reported as rank-biserial correlation (r) derived from the Wilcoxon Z, with 95% confidence intervals. Bootstrap resampling was applied to determine confidence intervals, with permutation-based *p*-values being used where applicable. Multiple comparisons were controlled using the Benjamini–Hochberg FDR procedure. Spearman’s rho correlation analysis was applied to examine relationships between continuous variables. Statistical significance was defined as *p* < 0.05.

## 3. Results

Forty-one patients were initially screened, 32 of whome were eligible and included in the analysis (16 men, 16 women). The patients’ mean age was 56.3 ± 19.2 years, with a median value of 60 (18–84) years. The right eye was treated in 53% of cases and the left eye in 47%. Preoperative BCVA was 0.28 ± 0.17 (median = 0.30; 0.02–0.60). Mean IOP was 12.8 ± 2.1 mmHg (median = 13; 10–18) and mean postoperative IOP was 15.4 ± 3.0 mmHg (median = 16; 11–22) (*p* = 0.001). In the YAG laser posterior capsulotomy application, the mean energy per shot was 3.15 ± 1.07 mJ (median = 3; 1.6–5), and the mean pulse count was 34.3 ± 20.4 (median = 29.5; 13–105). The total applied energy was recorded as 104.2 ± 61.4 mJ (median = 91.5; 32.5–290). The postoperative YAG laser interval was calculated at 8.65 ± 3.2 months (median = 8.5; 4–18) (Table 1). The most common complications were mild intraocular inflammation and minor glaucoma attacks, with no severe complications being observed.

A comparison of specular microscopy and corneal topography parameters before and after YAG laser posterior capsulotomy is presented in Table 2 and Table 3. Specular microscopy evaluation revealed no statistically significant change in endothelial cell count (NUM) after YAG laser application (*p* = 0.563). However, a significant decrease was observed in ECD, from 2184.05 ± 715.86 to 2057.2 ± 686.17 cells/mm^2^ (Δ = −126.9 cells/mm^2^; Δ% = −5.8%; *p* = 0.006). A statistically significant increase was observed in average cell area (AVG), from 529.25 ± 242.72 to 587.75 ± 281.09 µm^2^ (Δ = +58.5 µm^2^; Δ% = +11.1%; *p* = 0.004 A significant increase was also detected in minimum cell area (MIN), from 199.3 ± 170.62 to 248.35 ± 202.7 µm^2^ (Δ = +49.1 µm^2^; Δ% = +24.6%; *p* = 0.035). No statistically significant changes were determined in SD, CV, MAX, HEX, or CT parameters (*p* > 0.05).

Corneal topography analysis revealed a statistically significant decrease in anterior chamber angle (ACA) values, from 40.07 ± 10.34 to 35.42 ± 6.78 degrees (Δ = −4.65 degrees; Δ% = −11.6%; *p* = 0.048). No statistically significant changes were detected in ACD, ACV, or apex CCT parameters (*p* > 0.05).

**Figure 1:**
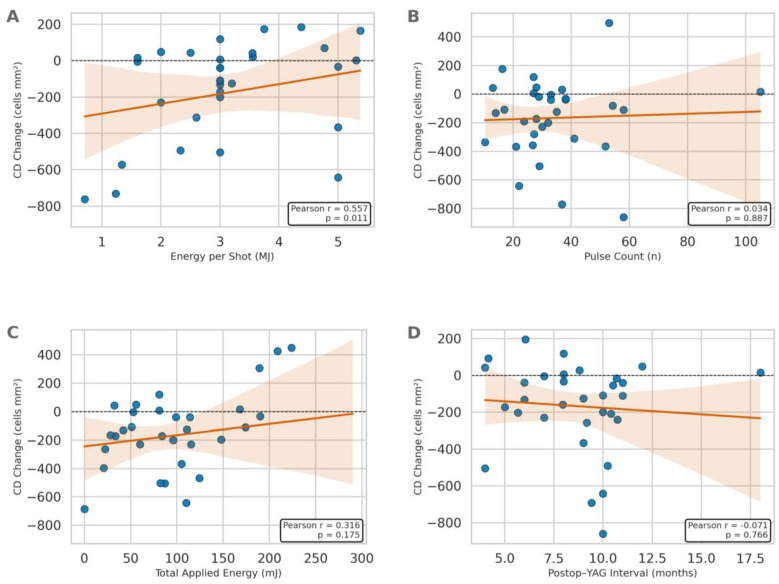
Correlation analysis between change in corneal endothelial cell density (CD Change, cells/mm^2^; Δ = post − pre) and clinical parameters: (**A**) Energy per shot (mJ), (**B**) Pulse count (n), (**C**) Total Applied Energy (mJ), (**D**) Postoperative YAG laser interval (months). Each blue dot represents an individual eye. Regression lines and Pearson correlation coefficients (r) with *p*-values are shown. The lines represent least-squares regression, and the shaded areas indicate the 95% confidence intervals. Only energy per shot exhibited a statistically significant positive correlation with CD change (r = 0.557, *p* = 0.011), suggesting less reduction (or greater preservation) in endothelial cell density at higher per-shot energies in the immediate postoperative period. Other parameters exhibited no significant association (all *p* > 0.05). Associations are cross-sectional and should be interpreted in the context of clinical energy titration and potential confounding.

The results of correlation analysis between age, BCVA, IOP, postoperative YAG laser interval, energy per shot, pulse count, total applied energy, and specular microscopy and corneal topography parameters are presented in Table 4. A statistically significant positive correlation was found between age and CV (r = 0.493; *p* = 0.027). Statistically significant positive correlations were observed between energy per shot and endothelial cell density (r = 0.557; *p* = 0.011) (Figure 1), average cell area (r = 0.544; *p* = 0.013), coefficient of variation (r = 0.446; *p* = 0.049), maximum cell area (r = 0.497; *p* = 0.026), and anterior chamber angle (r = 0.495; *p* = 0.026). In contrast, a statistically significant negative correlation was found between energy per shot and ACV (r = −0.659; *p* = 0.002). No statistically significant correlations were detected between intraocular pressure, postoperative YAG laser interval, pulse count, total applied energy parameters and specular microscopy or corneal topography parameters (*p* > 0.05).

## 4. Discussion

To the best of our knowledge, this study represents the first investigation of immediate corneal endothelial changes following YAG laser posterior capsulotomy, with measurements obtained one hour post-procedure. This temporal approach represents a fundamental departure from the existing literature and provides novel insights into the acute pathophysiology of laser-induced endothelial alterations.

One-hour early period measurements are of critical importance for evaluating the acute inflammatory response and sudden anatomical changes that develop after Nd:YAG laser capsulotomy. During this early period, the release of acute inflammatory mediators resulting from thermal effects and mechanical trauma caused by laser energy can lead to an increase in anterior chamber cells and protein concentration elevation. Furthermore, the sudden change in pressure balance between the vitreous cavity and anterior segment following the opening of the posterior capsule can cause temporary modifications in anterior chamber depth and angle. Evaluating these physiological processes within the first hour may be useful for the early detection of potential post-laser complications and may support timely management. However, due to the absence of longer-term follow-up, these implications should be regarded as preliminary.

The current literature on the effects of YAG laser capsulotomy on the corneal endothelium has predominantly focused on longer-term outcomes, with measurement time points ranging from one week to several months post-procedure. The existing literature on corneal endothelial changes following Nd:YAG laser posterior capsulotomy contains heterogeneous findings, with Paranjpe et al. reporting minimal endothelial cell loss (3 cells/mm^2^) in healthy corneas at one week (*p* = 0.78), while Samir et al. evaluated 60 patients (aged 18–65 years, mean 57.48 ± 8.1) using specular microscopy and documented a significant decrease in endothelial cell density from 1975.68 ± 582.7 to 1888.63 ± 580.8 cells/mm^2^ (a decrease of 87.05 cells/mm^2^ decrease, 4.4% *p* = 0.001). Eleiwa et al. showed substantial impairment (10.2% reduction) in Fucks endothelial corneal dystrophy patients at three months [15,16,17]. Critically, all existing investigations have involved follow-up periods ranging from one week to three months, with no study examining immediate post-procedural changes, representing a significant knowledge gap in our understanding of acute laser-induced endothelial alterations.

The results of this study revealed an immediate statistically significant decrease in endothelial cell density from 2184.05 to 2057.2 cells/mm^2^ within one hour of the procedure. This acute diminution is substantially greater than the minimal changes reported in previous studies at longer follow-up periods. This discrepancy suggests that significant endothelial recovery occurs between the immediate post-procedural period and the traditional measurement time points used in previous studies.

The acute decrease in endothelial cell density likely represents acute mechanical trauma from laser-induced shock waves and cavitation bubbles, capturing the direct physical impact before any recovery mechanisms occur. This decrease may reflect transient cellular dysfunction rather than permanent cell death, including temporary morphological changes, detachment, or functional impairment that resolves over time. Previous studies measuring at one week or later have missed this peak damage period, since the the corneal endothelium exhibits a remarkable regenerative capacity through cell migration and adaptation. The discrepancy between immediate and delayed measurements (minimal loss) indicates robust endothelial recovery capacity, suggesting that laser capsulotomy has a more significant acute impact than previously recognized while demonstrating the endothelium’s ability to recover from initial trauma.

In addition, the observed decrease in endothelial cell density at one hour post-procedure may be partially attributed to optical artifacts from contact lens and coupling gel use during Nd:YAG laser capsulotomy. These create additional optical interfaces, light scattering, and corneal surface alterations that compromise specular microscopy image quality and lead to artificially low cell density measurements, potentially explaining the discrepancy between immediate and long-term endothelial assessments. Crucial clinical information for identifying high-risk patients with pre-existing endothelial dysfunction such as Fuchs’ dystrophy, elderly patients, or those with compromised baseline endothelial cell counts, facilitates prompt intervention through early anti-inflammatory steroid therapy in cases of excessive endothelial trauma. It also permits real-time procedure optimization, including laser energy titration and technique modification, to minimize further endothelial compromise during subsequent treatments.

Determining whether the decrease in endothelial cell counts observed in this study reflects actual cell death or transient functional changes is of critical importance. Our findings reveal only acute-phase changes, and no definitive judgment regarding the permanence of these changes is possible. It should not be overlooked that some of the changes observed in the acute period may result from cellular edema, transient functional dysfunction, or measurement artifacts. A combination of long-term follow-up studies, functional evaluations, and morphological analyses is required to distinguish between them.

Mean intraocular pressure before Nd:YAG laser capsulotomy in this study was 12.8 mmHg, rising to 15.4 mmHg at one hour after the procedure. Paranjpe et al. reported that mean intraocular pressure increased from 13.50 ± 1.95 mmHg before the procedure to 13.86 ± 2.11 mmHg at one hour after treatment following Nd:YAG laser capsulotomy performed with 0.5–1.5 mJ energy and 6–15 shots [15]. Öztaş et al. measured intraocular pressure at 15.27 ± 3.05 mmHg in the preoperative period, rising to 15.57 ± 2.60 mmHg at the first week postoperatively and to 15.80 ± 2.19 mmHg at the first month [18]. However, this increase was not statistically significant (*p* = 0.47). Karahan et al. investigated the effect of capsulotomy size on intraocular pressure changes and determined a more pronounced increase in intraocular pressure in a large capsulotomy group compared to small capsulotomy group [19]. Although intraocular pressure declined to below preoperative levels at weeks 4 and 12 during the postoperative follow-up period in both groups, consistently higher values were recorded in the large capsulotomy group. Shetty et al. reported a significant increase in IOP following Nd:YAG laser posterior capsulotomy. Those authors reported that the increase in IOP was more pronounced with more than 40 pulses being applied during the procedure, and these patients were closely monitored [20]. Hassan et al. systematically evaluated intraocular pressure (IOP) changes at one hour following Nd:YAG laser posterior capsulotomy in 100 patients. The results demonstrated a significant elevation in mean IOP from 12.54 ± 2.35 mmHg preoperatively to 20.79 ± 6.38 mmHg at one hour post-procedure (*p* < 0.001), representing a statistically highly significant mean increase of 8.35 ± 5.52 mmHg [21]. The early postoperative IOP increase after Nd:YAG capsulotomy may be attributed to transient trabecular meshwork obstruction caused by laser debris and lens fragments, combined with inflammatory-mediated disruption of aqueous humor dynamics [22,23]. Moreover, the acute inflammatory response caused by laser energy increases aqueous humor viscosity, leading to an increase in protein concentration and the formation of cellular debris [20]. Channell et al. showed that all cases with post-capsulotomy intraocular pressure rises greater than 5 mmHg exhibited this elevation within the first 48 h following the procedure [24]. This transient pressure increase creates acute biomechanical stress on the corneal endothelium, triggering various adaptive and maladaptive responses at the cellular level.

These findings emphasize the critical need for intensive intraocular pressure monitoring following Nd:YAG laser capsulotomy, since post-procedural pressure elevations pose a significant risk for glaucomatous optic nerve damage. Enhanced surveillance protocols are essential, particularly in high-risk patients, to enable early detection and management of pressure spikes that may cause irreversible optic neuropathy. Additionally, prophylactic antiglaucomatous therapy should be considered in vulnerable patients to mitigate post-procedural pressure elevations and prevent acute glaucomatous episodes.

Corneal topography evaluation in this study revealed that while ACD exhibited no significant changes, a statistically significant decrease occurred in ACA following Nd:YAG capsulotomy. Pekel et al. evaluated iridocorneal angle changes following Nd:YAG laser posterior capsulotomy using manual measurement [25]. However, they detected no statistically significant differences in iridocorneal angle measurements at one hour postoperatively. In contrast, the present study demonstrated a significant decrease in anterior chamber angle using automated measurement algorithms. This discrepancy between the findings may be attributed to methodological differences between manual and automated measurement techniques, variations in patient population characteristics, or differences in measurement protocols and procedures. In contrast to our findings, Eliaçık et al.’s prospective study provides objective evidence of anterior segment remodeling following Nd:YAG laser capsulotomy in pseudophakic eyes. Using AS-OCT technology, those authors documented significant anterior chamber deepening (3.71 ± 0.11 mm to 3.77 ± 0.10 mm) and angle widening (nasal: 34.5 ± 1.67° to 35.51 ± 1.64°; temporal: 34.8 ± 1.55° to 36.17 ± 1.51°) by postoperative day 3, suggesting favorable anatomical changes that may have implications for intraocular pressure dynamics [26]. In a different study, Öztas et al. reported that anterior chamber angle measurements increased from 43.41 degrees at baseline to 45.56 degrees at one week and 44.56 ± 6.13 degrees at one month, this change being statistically significant [18]. The acute phase may involve sudden mechanical effects on the posterior capsule, potentially including vitreous movement and forward displacement of the lens–iris diaphragm complex, as suggested by previous reports. However, these mechanisms were not directly measured in the present study. These changes, as documented in our study, lead to the emergence of a temporary anterior chamber angle. This temporary narrowing, which is clinically significant and prolongs anatomical evaluations, may contribute to the pathophysiological mechanism underlying the transient increase in intraocular pressure frequently observed in the early post-capsulotomy period. Accordingly, future studies incorporating high-speed imaging or anterior segment OCT would be useful in terms of directly evaluating these proposed mechanisms.

Bhargava et al. demonstrated that energy requirements increase significantly with PCO severity [27]. This energy-intensity relationship reflects the underlying pathophysiological process in which lens epithelial cells undergo epithelial-mesenchymal transition, resulting in increased collagen deposition and fibrous tissue formation that require higher photodisruptive energy for effective ablation. Building upon this pathophysiological understanding, we investigated the correlations between increased energy requirements associated with PCO severity and corneal specular microscopy and topographic changes following Nd:YAG capsulotomy.

Analyses revealed statistical associations between energy per shot and the magnitude of acute endothelial and anterior segment changes. However, in the light of the study’s prospective observational design without randomization, these associations should not be interpreted as causal in nature. The findings are hypothesis-generating and warrant confirmation in controlled studies. Notably, although ECD decreased at the group level by approximately 5.8% postoperatively, energy per shot exhibited a positive cross-sectional correlation with ECD. This apparent discrepancy likely reflects clinical energy titration and confounding. Operators may adjust per-shot energy according to PCO characteristics and baseline ocular status, while total tissue burden is better captured by total energy and pulse count. Accordingly, the per-shot metric should be interpreted with caution in isolation. The robust negative correlation between energy per shot and ACV is consistent with an acute mechanical response (e.g., transient anterior displacement from shock waves and zonular tension), rather than inflammatory edema, in the light of its magnitude and immediate timing. The concomitant positive association with ACA further supports the notion of complex, geometry-driven changes in the anterior segment following higher-energy capsulotomy. Age-related effects were also evident, with older patients demonstrating greater endothelial size variability (positive correlation between age and CV). Clinically, the short-term effect sizes observed in this study were small-to-moderate for endothelial metrics and moderate for anterior segment geometry.

However, in the absence of longer-term follow-up and functional endpoints, it remains unclear whether the observed changes represent transient artifacts or clinically meaningful alterations, particularly due to the single within-hour time point, potential immediate post-procedure degradation of specular image quality, and the lack of longitudinal clinical outcomes. These findings should therefore be interpreted as preliminary and hypothesis-generating. Future studies should incorporate longitudinal follow-up with predefined functional endpoints, repeated high-quality imaging (e.g., anterior segment OCT and specular microscopy with image-quality metrics), and larger, ideally randomized cohorts to determine the durability, mechanisms, and clinical relevance of these within-hour changes.

## 5. Limitations

The principal limitation of this study is that only acute changes occurring within the first hour post-procedurally were evaluated. Another major limitation is the absence of a control group, which substantially limits causal inference and our ability to distinguish procedure-related changes from natural variability and measurement noise. The absence of longer-term outcomes prevents any assessment of the durability, clinical significance, or temporal course of laser effects and potential late complications. Any clinical implications should therefore be considered preliminary and hypothesis-generating. Additional limitations include the lack of quantitative capsulotomy diameter measurements, the absence of standardized posterior capsular opacification grading systems, a relatively small sample size limiting statistical power and generalizability, and the single-center design restricting external validity. Early measurement timing may have been influenced by acute inflammatory responses and corneal edema, potentially affecting specular microscopy accuracy. Mechanistic interpretations were not directly measured and should be considered speculative. Despite these limitations, this study offers preliminary insights into the immediate effects of Nd:YAG laser capsulotomy on anterior segment parameters and identifies energy-dependent associations. Additionally, specular measurements were limited to the central cornea; para-central fields were not acquired, precluding assessment of regional variation. Accordingly, our conclusions pertain to the central corneal region. Future studies should entail longer-term follow-up (minimum 3–6 months), larger multi-center cohorts, standardized multi-field imaging to evaluate spatial hetero-geneity, randomized controlled designs with appropriate control groups, and comprehensive assessment of both anatomical and functional outcomes (with pre-specified clinically meaningful thresholds) to determine durability and clin-ical relevance.

## 6. Conclusions

This study demonstrates that Nd:YAG laser posterior capsulotomy produces immediate energy-dependent changes in corneal endothelial cell density and anterior segment parameters within the first hour following treatment. However, these associations do not establish causality. While minimizing energy per shot may be reasonable, definitive protocol recommendations require confirmation by larger longitudinal and ideally randomized studies. The identification of significant correlations between laser energy levels and endothelial cell alterations represents a preliminary observation for optimizing treatment protocols in order to minimize corneal damage. Based on these findings, we recommend the implementation of standardized energy protocols, baseline endothelial assessment, and post-procedural monitoring to enhance patient safety. These results contribute valuable insights for developing individualized treatment approaches that balance therapeutic efficacy with corneal preservation in pseudophakic patients requiring capsulotomy.

## Figures and Tables

**Table 1:** Patient Demographics and YAG Laser Treatment Parameters.

Demographic Characteristics		n = 32 (%)	
**Gender**	Male	16 (50%)	
	Female	16 (50%)	
**Laterality**	Right	17 (53%)	
	Left	15 (47%)	
**Baseline characteristics**		**Mean ± SD**	**Median (Min-Max)**
Age (years)		56.3 ± 19.18	60 (18–84)
BCVA *		0.28 ± 0.17	0.3 (0.02–0.6)
IOP (mmHg)		12.8 ± 2.12	13 (10–18)
**Nd:YAG laser parameters**			
Energy per Shot (mJ)		3.15 ± 1.07	3 (1.6–5)
Postop-YAG Laser Interval (months)		8.65 ± 3.2	8.5 (4–18)
Pulse Count (n)		34.3 ± 20.37	29.5 (13–105)
Total Applied Energy (mJ)		104.19 ± 61.4	91.5 (32.5–290)

BCVA: (Best Corrected Visual Acuity * (decimal scale)), IOP: Intraocular Pressure, mJ: millijoules.

**Table 2:** A Comparison of Specular Microscopy and Parameters Before and After YAG Laser Capsulotomy.

Parameters	Before YAG Laser		After YAG Laser		Z	*p-*Value
	Mean ± SD	Median (Min-Max)	Mean ± SD	Median (Min-Max)		
**IOP (mmHg)**	12.8 ± 2.12	13 (10–18)	15.4 ± 3.0	16(11–22)	−3.29	**0.001**
**Specular Microscopy**						
NUM (n)	112.65 ± 56.39	116.5 (18–182)	116.05 ± 54.81	119 (11–208)	−0.579	0.563
CD (cells mm^2^)	2184.05 ± 715.86	2307.5 (821–3176)	2057.2 ± 686.17	2186 (788–3095)	**−2.763**	**0.006**
AVG (µm^2^)	529.25 ± 242.72	433.5 (297–1218)	587.75 ± 281.09	461 (323–1269)	**−2.912**	**0.004**
SD (µm^2^)	152.9 ± 82.4	152.5 (64–460)	152.5 ± 59.76	148.5 (60–309)	−0.168	0.867
CV (%)	30.6 ± 7.29	30.5 (18–44)	28.9 ± 5.52	29.5 (18–37)	−0.963	0.335
MAX (µm^2^)	1185.3 ± 348.85	1203 (730–1961)	1372.4 ± 559.37	1306 (699–2797)	−1.755	0.079
MIN (µm^2^)	199.3 ± 170.62	140 (42–662)	248.35 ± 202.7	146.5 (42–763)	**−2.110**	**0.035**
HEX (%)	63.6 ± 7.84	64 (44–76)	65.55 ± 6.81	65 (46–75)	−1.135	0.256
CT (µm)	530.75 ± 20.89	529.5 (498–577)	527.5 ± 20.06	528 (484–560)	−0.747	0.455

NUM: Number of Analyzed Cells, CD: Cell Density, AVG: Average Cell Area, SD: Standard Deviation of Cell Area, CV: Coefficient of Variation of Cell Area, MAX: Maximum Cell Area, MIN: Minimum Cell Area, HEX: Hexagonality, CT: Central Corneal Thickness, The Wilcoxon Signed Ranks Test was used to compare pre- and post-measurements of continuous variables. Bold: They are statistically significant values so they are in bold. Statistical significance was defined as *p* < 0.05.

**Table 3:** Comparison of Anterior segment parameters in corneal topography Before and After Nd:YAG Laser Capsulotomy.

Corneal Topography Parameters	Before YAG Laser		After YAG Laser		Z	*p-*Value
	Mean ± SD	Median (Min-Max)	Mean ± SD	Median (Min-Max)		
ACD (mm)	4.48 ± 0.46	4.47 (3.67–5.26)	4.25 ± 0.74	4.28 (2.02–5.17)	−1.751	0.080
ACA (degrees)	40.07 ± 10.34	38.23 (20.7–68.7)	35.42 ± 6.78	35.26 (22.3–47.3)	**−1.979**	**0.048**
ACV (mm^3^)	183.5 ± 31.78	182.5 (121–236)	183.5 ± 29.17	184 (129–228)	−0.087	0.931
Apex CCT (µm)	527.6 ± 20	523 (505–570)	522.6 ± 16.63	519.5 (501–560)	−1.833	0.067

ACD: Anterior Chamber Depth, ACA: Anterior Chamber Angle, ACV: Anterior Chamber Volume, Apex CCT: Central Corneal Thickness at the Corneal Apex. The Wilcoxon Signed Ranks Test was used to compare pre- and post-measurements of continuous variables. Statistical significance was defined as *p* < 0.05.

**Table 4:** Correlation analysis of baseline clinical variables and Nd:YAG laser parameters with ocular metrics derived from specular microscopy and corneal topography.

		**NUM**	**CD**	**AVG**	**SD**	**CV**	**MAX**	**MIN**	**HEX**	**CT**	**ACD**	**ACA**	**ACV**	**CCT**
**Age (years)**	r	−0.035 0.882	0.428 0.060	0.403 0.078	0.206 0.384	0.493 0.027	0.332 0.153	0.477 0.034	0.089 0.710	0.431 0.058	0.067 0.779	0.242 0.304	−0.293 0.210	−0.323 0.165
	*p*													
**BCVA** *	r	−0.153	−0.291	−0.313	−0.333	−0.272	−0.364	−0.446	−0.269	−0.116	0.120	−0.298	0.457	0.277
	*p*	0.520	0.214	0.179	0.151	0.055	0.115	0.049	0.251	0.625	0.614	0.203	0.053	0.237
**IOP (mmHg)**	r	−0.191	−0.226	−0.236	−0.172	−0.106	0.100	−0.335	−0.466	0.159	−0.045	−0.020	0.109	0.078
	*p*	0.421	0.339	0.316	0.469	0.657	0.674	0.149	0.038	0.504	0.851	0.934	0.647	0.744
**Postop-YAG Laser Interval (months)**	r	0.089	−0.071	0.012	0.381	0.516	−0.104	0.295	−0.028	0.067	0.020	−0.046	−0.009	−0.001
	*p*	0.708	0.766	0.960	0.098	0.020	0.663	0.207	0.905	0.780	0.932	0.847	0.970	0.997
**Energy per Shot (mJ)**	r	0.017	0.557	0.544	0.032	0.446	0.497	0.314	0.390	0.266	0.015	0.495	−0.659	0.132
	*p*	0.943	0.011	0.013	0.892	0.049	0.026	0.177	0.089	0.258	0.949	0.026	0.002	0.578
**Pulse Count (n)**	r	0.160	0.034	0.082	0.004	0.068	−0.331	0.196	0.103	0.371	0.004	−0.042	0.015	0.254
	*p*	0.499	0.887	0.731	0.987	0.776	0.154	0.408	0.665	0.107	0.987	0.860	0.951	0.280
**Total Applied Energy (mJ)**	r	0.129	0.316	0.346	0.007	0.371	−0.007	0.364	0.332	0.445	−0.011	0.156	−0.271	0.312
	*p*	0.587	0.175	0.135	0.977	0.108	0.977	0.115	0.152	0.050	0.962	0.510	0.248	0.180

NUM: Number of Analyzed Cells. CD: Cell Density. AVG: Average Cell Area. SD: Standard Deviation of Cell Area. CV: Coefficient of Variation of Cell Area. MAX: Maximum Cell Area. MIN: Minimum Cell Area. HEX: Hexagonality. CT: Central Corneal Thickness. ACD: Anterior Chamber Depth. ACA: Anterior Chamber Angle. ACV: Anterior Chamber Volume. Apex CCT: Central Corneal Thickness at the Corneal Apex. Spearman’s rho correlation analysis was performed to examine the relationship between continuous variables. * means decimal scale. BCVA means Best Corrected Visual Acuity. Bold: They are the headlines. We made them bold to separate them easily from the other parameters in the table. Statistical significance was defined as *p* < 0.05.

## Data Availability

The original contributions presented in this study are included in the article. Further inquiries can be directed to the corresponding author.
